# Bilateral Ischaemic Stroke in Eosinophilic Granulomatosis With Polyangiitis

**DOI:** 10.7759/cureus.96427

**Published:** 2025-11-09

**Authors:** Mostafa Abdelati, Vruti Patel, Islam I Zaid

**Affiliations:** 1 Department of Stroke Medicine, King's College Hospital NHS Foundation Trust, London, GBR

**Keywords:** asthma, bilateral stroke, cns involvement, eosinophilic granulomatosis with polyangiitis (egpa), ischaemic stroke, vasculitis

## Abstract

A woman in her seventies with a recent diagnosis of asthma presented with right-sided limb weakness and progressive neurological decline. Magnetic resonance imaging of the brain revealed bilateral ischaemic infarcts in watershed territories. Further clinical deterioration prompted extensive investigations, which demonstrated multisystem involvement, including the lungs, heart, kidneys, and skin. A diagnosis of eosinophilic granulomatosis with polyangiitis (EGPA) was made. The patient was started on immunosuppressive therapy and antiplatelet agents, leading to significant clinical improvement. Although central nervous system involvement can occur in EGPA, ischaemic stroke remains a rare manifestation. This case highlights the importance of considering systemic vasculitis in patients presenting with stroke and multisystem features.

## Introduction

Eosinophilic granulomatosis with polyangiitis (EGPA) is a rare form of systemic necrotizing vasculitis that is frequently associated with antineutrophil cytoplasmic antibodies (ANCA). It is one of the three primary subtypes of ANCA-associated vasculitis, along with granulomatosis with polyangiitis (GPA) and microscopic polyangiitis (MPA) [1]. EGPA predominantly affects the respiratory tract, typically presenting with asthma and sinusitis, followed by systemic involvement resulting from eosinophilic infiltration. This can lead to the involvement of the cardiovascular system, gastrointestinal tract, peripheral nervous system, and skin [2,3]. Due to its broad spectrum of clinical manifestations, early recognition of EGPA can be challenging. Central nervous system involvement is uncommon, occurring in approximately 5-9% of cases, and is associated with a poorer prognosis [2,3]. Diagnosis can be challenging due to its rarity and overlap with other eosinophilic disorders. The 2022 American College of Rheumatology/European Alliance of Associations for Rheumatology (ACR/EULAR) classification criteria for EGPA recommend a points-based system, with a score of ≥6 supporting classification [4]. The diagnosis should be based on a combination of clinical findings, laboratory markers, imaging, and histopathological evidence.

Thromboembolic events in EGPA may occur due to eosinophil-mediated endothelial injury, hypercoagulability, or cardiac involvement-even without a clear embolic source [5]. We report a patient who initially presented with confusion and right-sided limb weakness, graded as Medical Research Council (MRC) 4/5 [6], and a National Institutes of Health Stroke Scale (NIHSS) score of two [7]. Over the subsequent days, her condition deteriorated, with evidence of multisystem involvement. Laboratory investigations revealed eosinophilia, and neuroimaging demonstrated bilateral ischaemic infarcts. Further diagnostic evaluation established the diagnosis of EGPA. The patient was commenced on immunosuppressive therapy, resulting in marked clinical and radiological improvement.

## Case presentation

A woman in her seventies of Asian descent had a longstanding history of type 2 diabetes, hypertension, and hypercholesterolemia, all diagnosed 16 years earlier. She was diagnosed with adult-onset asthma 12 months prior to presentation and commenced on preventive inhaler therapy. Her asthma remained stable initially, but over recent months she required multiple courses of corticosteroids and antibiotics for recurrent chest infections and sinusitis.

In the weeks before presentation, she had multiple emergency department visits with predominantly respiratory symptoms and nonspecific complaints, including fatigue, musculoskeletal pain, fever, acid reflux, headache, abdominal pain, and constipation. Approximately two days before hospital admission (day 0), the patient developed a new non-blanching purpuric rash on both legs, and her family noticed increasing confusion. On admission (day two), she developed right-sided limb weakness, prompting a stroke alert. Examination revealed MRC grade 4/5 in the right upper and lower limbs, with mild confusion but orientation to time, place, and person. NIHSS score was two, reflecting right-sided drift. Vital signs were within normal limits, including blood pressure, oxygen saturation, heart rate, respiratory rate, and blood glucose. Chest auscultation was clear, and she was noted to have a low-grade fever. A non-contrast CT and CT angiogram scan of the head were reported as normal.

Differential diagnoses included meningoencephalitis, atypical fungal infection, stroke with atypical features, and ANCA-associated vasculitis. Empirical intravenous antibiotics were initiated for possible central nervous system infection, and aspirin 300 mg was administered pending MRI. Given the wide differential diagnoses, Investigations were extended to include lumbar puncture, ANCA panel, complement levels (C3/C4), fungal markers (Beta-D-glucan), viral serologies (HIV, hepatitis B and C, EBV, CMV), antiphospholipid antibody screening, travel history assessment, and CT of chest, abdomen, and pelvis. Lymphoma was also considered as a differential diagnosis given the clinical presentation. The only positive finding was an elevated anti-myeloperoxidase (anti-MPO) antibody, supporting the diagnosis of EGPA.

On day three, the patient’s neurological status deteriorated, with worsening right-sided weakness (MRC grade 0/5 in the upper limb, and 3/5 lower limb), new right facial weakness, reduced speech output, and Glasgow Coma Scale (GCS) 13/15. Repeat head CT was normal. By day five, she required high dependency unit admission due to progressive neurological decline and respiratory compromise. On day six, repeat head imaging revealed multifocal strokes, and the MRI demonstrated bilateral watershed and cerebellar infarcts.

A renal biopsy was recommended but deferred due to respiratory instability. The patient spent 10 days in a specialized intensive care unit before being transferred to a medical ward, and later to a stroke rehabilitation unit following clinical improvement.

Investigations and treatment

Laboratory investigations (Table 1) on admission revealed marked peripheral eosinophilia (12.35 × 10⁹/L). Retrospective review of prior blood tests showed mildly elevated eosinophil counts over several months, though not previously high enough to prompt further investigation.

**Table 1:** Key laboratory results on hospital admission (day two) and at six-month follow-up Summary of the patient's relevant laboratory findings at initial presentation (peak values) and after six months of treatment. Improvement in inflammatory markers and normalization of eosinophil count is noted. MPO: myeloperoxidase; PR3: proteinase 3; NT-proBNP: N-terminal pro-B-type natriuretic peptide

Laboratory tests	On admission	After 6 months	Reference range
White cell count	21 x 10⁹/L	5.9 x 10⁹/L	2.9 - 9.6 x 10⁹/L
Eosinophils	12.35 × 10⁹/L	0.17 × 10⁹/L	0.00 - 0.40 x 10⁹/L
C-reactive protein	131 mg/L	1 mg/L	<5 mg/L
Anti-MPO antibodies	>134.0 IU/mL	16 IU/mL	<3.5 IU/mL
Anti-PR3 antibodies	<0.2	<0.2	<2.0 IU/mL
Troponin I	8,000 ng/L	16 ng/L	<16 ng/L
Troponin T	2309 ng/L	38 ng/L	≤14 ng/L
NT-proBNP	10331 ng/L	404 ng/L	<400 ng/L
Urine protein/creatinine ratio	108 mg/mmol	62.5 mg/mmol	0.0 - 15.0 mg/mmol
Creatinine	48 µmol/L	48 µmol/L	49 - 90 µmol/L
Urea	3.8 mmol/L	4.8 mmol/L	2.5 - 7.8 mmol/L

Renal involvement was evident through an elevated urine protein-creatinine ratio (UPCR), although serum creatinine remained within normal limits. Urinalysis revealed microscopic haematuria and proteinuria. Cardiac investigations revealed significant myocardial involvement. Serum troponin I and T levels were markedly elevated, while electrocardiogram (ECG) and transthoracic echocardiography were within normal limits (ejection fraction 60%). Cardiac magnetic resonance imaging (MRI) demonstrated diffuse subendocardial fibrosis consistent with endomyocarditis secondary to a vasculitic process.

**Figure 1:**
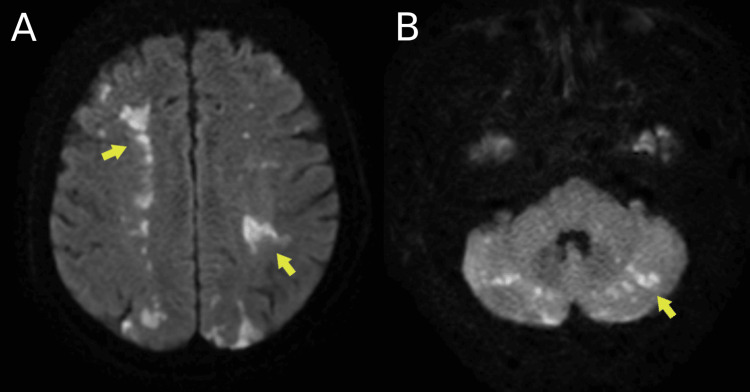
MRI brain (DWI) on day six Diffusion-weighted imaging (DWI) demonstrates hyperintense lesions (yellow arrows), indicating acute infarcts in bilateral watershed territories. Infarcts are visible in (A) the frontoparietal lobes and (B) cerebellum, consistent with multifocal ischaemic injury.

Neurological imaging confirmed multifocal ischaemic injury. Brain MRI on (day six) demonstrated multiple areas of diffusion restriction on diffusion-weighted imaging (DWI), indicating acute infarcts in the bilateral cerebellar hemispheres, basal ganglia, and watershed cortical territories (Figure 1). A repeat non-contrast CT head performed 48 hours later revealed corresponding ischaemic changes.

**Figure 2:**
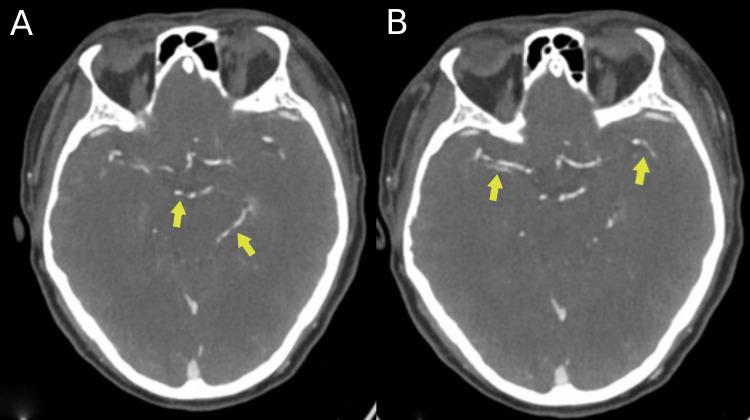
CT angiography of intracranial vessels CT angiogram reveals segmental beaded vascular irregularities, noted in the posterior cerebral arteries (A) and in the middle cerebral arteries, particularly in the M2 branches (B). These findings in our patient's clinical presentation and radiological findindgs were suggestive of vasculitis.

CT angiography of the head and neck, which was performed on admission, was later reviewed at a multidisciplinary neuroradiology meeting, demonstrated segmental irregularities in the middle and posterior cerebral arteries. These findings, when considered in the context of systemic features and serology, were consistent with cerebral vasculitis (Figure 2).

**Figure 3:**
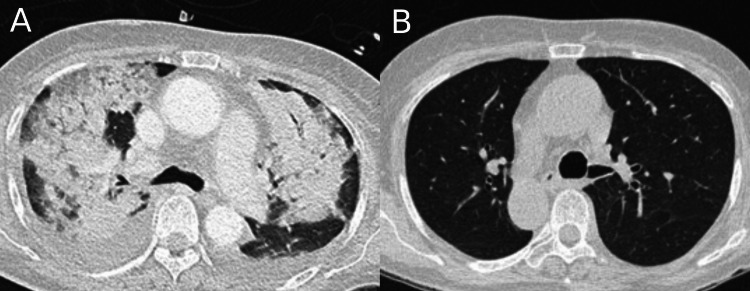
CT chest on day seven post admission (A) and three weeks after treatment initiation (B) (A) CT chest on day seven demonstrates extensive bilateral consolidation and pleural effusions, consistent with pulmonary involvement in EGPA. (B) Follow-up CT chest three weeks after initiation of immunosuppressive therapy shows marked improvement, with resolution of consolidation and significant reduction in pleural effusions.

CT chest showed bilateral pulmonary infiltrates with consolidation, moderate pleural effusions, and radiological evidence of pulmonary haemorrhage (Figure 3A), consistent with pulmonary involvement in EGPA.

A diagnosis of EGPA was established based on clinical, serological, and radiological findings. Due to the severity of presentation, immunosuppressive therapy was initiated within 48 hours of admission. Initial treatment included intravenous methylprednisolone 500 mg once daily for three days, followed by high-dose oral prednisolone 60 mg, tapered immediately over two months. The patient also received five sessions of plasma exchange and five doses of cyclophosphamide administered at two-week intervals for remission induction.

Marked clinical and radiological improvement was observed following treatment, with eosinophil count normalizing within three days and near-complete resolution of pulmonary findings on follow-up CT (Figure 3B). The neurorehabilitation process included balance training and range-of-motion exercises. By three months, she was able to use a wheelchair, and over time progressed to walking with a frame. After six months of hospitalization, she was discharged with significant neurological recovery, improved cognitive function, and mobility using a walking stick, requiring minimal assistance for daily activities.

The patient is currently on methotrexate 15 mg once weekly for remission maintenance. The addition of rituximab, a B-cell-depleting agent, is under consideration to reduce the risk of relapse. For secondary stroke prevention, low-dose aspirin (75 mg daily) was our choice after a multidisciplinary discussion, considering her bleeding risk and other cardiovascular risk factors.

## Discussion

Antineutrophil cytoplasmic antibody (ANCA)-associated vasculitides (AAV) are autoimmune diseases causing necrotizing inflammation of small-to-medium vessels, affecting multiple organs. The main subtypes include granulomatosis with polyangiitis (GPA), microscopic polyangiitis (MPA), and eosinophilic granulomatosis with polyangiitis (EGPA) [1,8]. GPA features necrotizing granulomatous inflammation of the respiratory tract and vasculitis with glomerulonephritis. MPA involves necrotizing vasculitis without granulomas, often with pulmonary capillaritis and glomerulonephritis. EGPA is characterized by eosinophil-rich granulomatous inflammation, asthma, eosinophilia, and MPO-ANCA positivity, especially with glomerulonephritis [1,8]. Subclassification by antigen helps diagnosis: GPA is linked to PR3-ANCA, while MPA and about 40% of EGPA cases associate with MPO-ANCA [1].

Diagnosing EGPA remains challenging due to its rarity, with a global prevalence estimated at 15.27 cases per million people [9] and its heterogeneous presentation, which can delay recognition. EGPA commonly affects the lungs, skin, heart, and kidneys. Although peripheral nervous system involvement is frequently observed (55-72%), central nervous system (CNS) involvement is much less common, occurring in only 5-9% of cases [3]. When CNS involvement does occur, ischaemic stroke is the most reported presentation, whereas intracerebral haemorrhage is associated with worse outcomes [10]. The 2022 American College of Rheumatology (ACR) classification criteria for EGPA recommend assigning points based on clinical and laboratory findings. A score of ≥6 supports a diagnosis of EGPA. Criteria include eosinophil count ≥1×10⁹/L (+5 points), obstructive airway disease (+3), nasal polyps (+3), extravascular eosinophilic-predominant inflammation (+2), and mononeuritis multiplex or motor neuropathy not due to radiculopathy (+1) [4]. Our patient met the criteria with a total score of 8, including 5 points for a high eosinophil count (≥1×10⁹/L) and 3 points for obstructive airway disease, thereby supporting the diagnosis. However, it is important to note that these classification criteria are not diagnostic tools but rather aids for disease classification. The final diagnosis should be based on a combination of clinical findings, laboratory markers, imaging, and histopathological evidence where feasible [11].

Watershed (border-zone) infarcts, as observed in our patient, are ischaemic lesions that occur at the junctions of cerebral arterial territories. These regions are more susceptible to hypoperfusion and infarction due to their distal location in the vascular supply. While classically associated with systemic hypotension and hypoperfusion [12], alternative mechanisms may contribute to EGPA. Hypereosinophilia itself has been implicated in endothelial dysfunction and thrombotic events due to increased blood viscosity and the release of cytotoxic eosinophilic granules [5,13]. The "impaired washout" theory hypothesizes that low-flow regions like watershed zones are less capable of clearing eosinophilic toxins, thereby increasing susceptibility to thrombosis [14]. These proposed mechanisms may act synergistically to result in cerebrovascular events such as ischaemic stroke in patients with EGPA. 

Although no definitive cardiac source of embolism was identified in our patient-based on ECG, Holter monitoring, and echocardiography-stroke was likely multifactorial. Similar case reports have described multiple thromboembolic events in EGPA patients, for which anticoagulation has been used successfully [15,16]. In another reported case, autopsy findings revealed a cardiac thrombus accompanied by eosinophilic infiltration and vasculitis, suggesting that cardiac embolism may contribute to infarction in patients with EGPA [17]. Our patient was managed conservatively with single antiplatelet therapy, balancing bleeding risk against thrombotic potential. Notably, no recurrent thromboembolic events were observed during hospitalization and early follow-up.

## Conclusions

This case highlights eosinophilic granulomatosis with polyangiitis (EGPA) as a rare but important differential diagnosis in patients presenting with acute neurological symptoms and multisystem involvement, particularly in the presence of eosinophilia. Central nervous system manifestations, such as bilateral watershed infarcts, may occur early in the course of EGPA and can contribute to diagnostic delays. Early recognition, a multidisciplinary approach, and timely initiation of immunosuppressive therapy are critical for achieving significant clinical improvement.
